# Pregnant women’s knowledge and needs on labor mobility: implications for childbirth preparation – a qualitative study

**DOI:** 10.1186/s12884-026-09316-6

**Published:** 2026-07-07

**Authors:** Marlene Lopes, Margarida Vieira, Alexandrina Cardoso

**Affiliations:** 1 Health Sciences Research Unit: Nursing, Nursing School of University of Coimbra, Coimbra, Portugal; 2 Faculty of Health Sciences and Nursing, Universidade Católica Portuguesa, Centre for Interdisciplinary Research in Health, Porto, Portugal; 3 Center for Health Technology and Services Research, Nursing School of University of Porto, Porto, Portugal

**Keywords:** Prenatal education, Childbearing women, Decision-making, Active birth, Upright positions

## Abstract

**Objective:**

To explore pregnant women’s knowledge, perceptions, and needs regarding mobility and upright positions during pregnancy, within the context of a childbirth preparation program.

**Methods:**

A qualitative study using an interpretive thematic approach was conducted in primary healthcare settings in Portugal between December 2023 and January 2024. Data were collected through group and individual semi-structured interviews, and analyzed using interpretive thematic analysis.

**Results:**

Twenty-three pregnant women from five Community Care Units participated. Three key themes were identified: (1) Living my own childbirth, (2) Stay active for a positive experience, and (3) Empowering myself for labor and birth. Women described childbirth as a personal and autonomous process, valuing a sense of control and active participation. Mobility during labor was understood as an important strategy for comfort and supporting labor progression. Participants expressed a desire to feel empowered through access to evidence-based information and opportunities to develop practical skills, which contributed to building confidence and anticipating participation in decision-making. However, women also recognised that institutional and organisational constraints within clinical environments could limit their ability to act on these preferences, potentially leading to frustration and dissatisfaction.

**Conclusion:**

Childbirth preparation programs should move beyond information provision to include strategies that strengthen women’s self-efficacy and support their capacity to participate in decision-making, particularly regarding mobility during labor. At the same time, hospital environments need to be aligned with these preparatory efforts by respecting women’s choices and supporting mobility and active participation during labor. These findings highlight the need for further research on interventions that support women’s decision-making processes, particularly in less flexible or highly medicalised healthcare settings.

## Introduction

While many women express a desire to actively participate in decisions about their pregnancy and childbirth [1], it is uncertain whether they are making fully informed choices that contribute to their self-efficacy and lead to a more positive birth experience [2, 3]. Decision-making around birth choices is complex and shaped by expectations that develop throughout pregnancy, influenced by women’s views, beliefs, interpretations, and biases. These factors help explain why certain options are favored over others. Decision-making in childbirth can be viewed as a continuum, with behaviors shaped by beliefs that frame childbirth as primarily physiological, medical, or a combination of both, all of which significantly influence women’s decisions [4].

On the other hand, intrapartum care, even in low-risk cases, is often shaped by a risk-averse mindset, driven by institutional risk management, fear of adverse outcomes, and personal values surrounding physiological childbirth. This perspective may explain the increasing rates of intervention [5]. Some healthcare professionals adopt a selective and paternalistic approach to information, offering limited options and leading women to “agree” with recommended interventions, rather than making truly informed decisions [5, 6]. A study conducted in Ireland by O’Brien et al. [7] revealed that contextual and relational factors, such as pressure to accept interventions and perceiving healthcare professionals as rushed or unavailable, can undermine women’s autonomy in decision-making during labor. These findings underscore the need to address resource limitations, time constraints, and systemic pressures that influence decision-making during labor. Valuing women’s autonomy and ensuring their informed, satisfactory decisions should be key indicators of quality obstetric care.

Movement and upright positions during labor have been consistently shown to offer significant benefits for both the childbirth process and outcomes. A Cochrane meta-analysis of 25 trials found that upright positions are associated with a shorter first stage of labor, a reduced incidence of cesarean sections, and fewer newborn admissions to neonatal intensive care units [8]. Additional reviews and meta-analyses have linked upright positions to a shorter second stage of labor, fewer episiotomies, and a reduction in abnormal fetal heart rate patterns [9, 10]. Increasing evidence also suggests that mobility and upright positions enhance the overall childbirth experience for women [2, 11]. In line with this body of evidence, the World Health Organization recommends that women be encouraged to stay mobile and adopt positions they find comfortable, including upright ones, during labor [1].

Despite this substantial evidence, recumbent positions continue to be the norm in many clinical settings, a practice influenced by a complex interplay of factors [2, 12–16]. In European hospitals, most women adopt horizontal positions based on healthcare professionals’ advice rather than personal preference [2, 16]. This discrepancy between preference and practice highlights a broader issue concerning women’s autonomy during labor.

Evidence shows that participation in Childbirth Preparation Programs can lead to more positive birth experiences, reduced intervention rates, increased use of non-pharmacological methods, greater involvement in decision-making, and enhanced feelings of self-efficacy and control during labor [17–19]. However, it has been observed that women do not always apply what they have learned, and it remains unclear which information they retain and prioritize in their decision-making. Many women report that, despite being informed about the benefits of freedom of movement, they lacked the necessary support or appropriate conditions to adopt an upright position during labor [2, 12]. Similarly, a study in Spain found that the primary reason women did not present their birth plans to healthcare professionals was that they were not prompted to do so [20]. This highlights the role of perceived social expectations in shaping women’s decisions, which can, in turn, affect their autonomy and satisfaction with the birth experience. It is therefore essential to actively consult women about their preferences and provide the necessary support throughout the childbirth process.

Numerous studies advocate for promoting maternal mobility during labor, focusing on educating midwives and addressing barriers within healthcare systems [14, 15]. Additionally, research has explored women’s perceptions of their mobility during labor [2]. A woman’s confidence and belief in her ability to give birth often begins during pregnancy and childbirth preparation. These factors shape her preferences for specific birthing positions and her response to midwives’ encouragement to adopt upright positions, ultimately influencing labor outcomes [21]. Given these considerations, this study aimed to explore pregnant women’s knowledge, perceptions, and needs regarding mobility and upright positions as they prepared to participate in a Childbirth Preparation Program. The findings may offer valuable insights for midwifery interventions, supporting women in making informed decisions about their birth during childbirth preparation.

## Methods

### Design

A qualitative descriptive study using an interpretive thematic analysis was conducted to explore the knowledge, perceptions, and needs of pregnant women regarding mobility and upright positions during labor, in the context of participation in a Childbirth Preparation Program within primary healthcare settings. This approach was considered appropriate to gain an in-depth understanding of women’s perspectives and meanings attributed to labor mobility, while remaining close to participants’ accounts and experiences.

Data were gathered through four group interviews and three individual interviews, using semi-structured questions, between December 2023 and January 2024. The study adhered to the Standards for Reporting Qualitative Research (SRQR) guidelines [22].

Ethical approval was obtained from the Health Ethics Committee of the Central Regional Health Administration on June 22, 2023 (no. 52/2023). Participants were provided with comprehensive information about the study, informed of their right to refuse or discontinue participation at any point, and provided informed consent through an online form.

### Setting, sampling, participants and recruitment

Seven Community Care Units (CCUs) located in the central region of Portugal, each offering a Childbirth Preparation Program, were selected as focal points for the initial phase of a comprehensive investigation. In Portugal’s National Health Service, women with low-risk pregnancies are primarily followed in primary care by family physicians, while obstetricians provide care in higher-risk pregnancies and in hospital-based maternity units. In Portugal, the professional role internationally referred to as “midwife” is undertaken by registered nurses who complete a two-year master’s programme in Maternal and Obstetric Health Nursing after their initial nursing degree. Therefore, the term “nurse-midwife” is used throughout this manuscript to reflect the Portuguese professional title and context. Nurse-midwives play a key role in antenatal education, childbirth preparation, and intrapartum care. Most CCUs have a nurse-midwife and offer free childbirth education to all expectant couples who seek it. All maternity units are led by obstetricians, with varying institutional practices related to mobility and upright positions during labor.

The overarching aim of this research was to develop and evaluate an intervention designed to empower women for active childbirth in a pilot study. To enhance representativeness and ensure geographic, rural–urban, and cultural diversity, the selection process involved intentional sampling to recruit pregnant women from these seven CCUs. The inclusion criteria included women about to begin the program, aged 18 years or older, and experiencing a low-risk pregnancy. Women were eligible regardless of parity. Women with pregnancy-related complications were excluded. Additional exclusion criteria included not understanding or speaking Portuguese. Nurse-midwives from the CCUs contacted eligible participants, who, upon consent, shared their email and phone numbers with the research leader. The research leader then contacted 46 women from five CCUs, and 23 agreed to participate.

### Data collection

Group interviews were initially planned as the primary data collection method, as they allowed interaction and shared reflection among participants. However, when women were unable to attend the scheduled group sessions but remained interested in participating, individual interviews were conducted to enable their participation. All interviews were conducted via Zoom by the lead researcher and scheduled in consultation with participants, in order to facilitate participation across geographically diverse regions and reduce logistical barriers [23].

Group and individual interviews followed the same semi-structured guide and explored the same core topics. The interviews followed a semi-structured guide designed to respect participants’ personal and cultural contexts while avoiding leading questions, thereby fostering rich and contextualised narratives (Table 1). The script was pre-tested with two pregnant women who did not participate in the final study, leading to minor refinements in question wording [24]. Before the interviews, all participants completed an online sociodemographic and obstetric form. Interviews continued until interpretive saturation was reached, meaning that no new meanings relevant to the study aim emerged [25]. All interviews were audio-recorded and transcribed verbatim by the lead researcher.

**Table 1:** Guiding questions for semi-structured interviews

Questions
1. How do you envision your experience during childbirth?
2. What are your thoughts or knowledge regarding the freedom of movement, various positions, or upright postures during labor? Do you believe these factors will have any impact?
3. What information are you interested in learning about mobility and upright positions during labor? How would you prefer to receive this knowledge, and in what ways would you like to develop the skills necessary to incorporate them into your birthing experience?
4. In what ways do you believe gaining more insight would enhance your confidence in making decisions about your childbirth?

### Data analysis

Interview transcripts were analysed through an interpretive thematic analysis aimed at understanding how women made sense of mobility and upright positions during labor [26].

First, ML read all transcripts in full and reviewed the video recordings to become familiar with each participant’s account. During this phase, initial notes were written to capture key ideas, expressions, and issues raised by participants, without applying a predefined coding framework. In a second phase, segments of text related to women’s understandings, expectations, and needs regarding labor mobility were identified and grouped, allowing recurrent ideas to emerge across interviews. These groupings were progressively discussed with AC, and similar meanings were clustered and refined into preliminary themes. Throughout the analysis, the researchers moved back and forth between interviews and the developing themes, revisiting the transcripts to ensure that interpretations remained grounded in participants’ accounts.

NVivo software was used to organise transcripts and support the management and retrieval of text segments during analysis. Both authors were involved in discussing and refining the themes, and interpretive decisions were reached through dialogue. Member checking was not conducted; however, credibility was supported through prolonged engagement with the data, collaborative interpretation, and transparency in the analytic process.

### Reflexivity

Recognizing the potential impact of researchers’ perspectives on questioning, interpretation, and the co-creation of meaning is crucial in qualitative research [27]. Therefore, it is essential to acknowledge and articulate positionality throughout the research process. For transparency, both ML and AC are nurse-midwives. ML is a PhD student with extensive experience in antenatal education and childbirth support, while AC holds a PhD in Nursing and is a professor actively involved in researching the care provided by nurse-midwives. Both share a commitment to women-centered care, which they consider fundamental, and advocate for women’s autonomy in health-related decision-making as a pathway to enhancing positive childbirth experiences.

## Results

The study included 23 women from five Community Care Units (CCUs), aged 27 to 41 years (M = 33.83, SD = 4.58), with gestational ages from 26 to 33 weeks (M = 29.57, SD = 2.71). Four had completed high school, 9 held bachelor’s degrees, 8 had master’s degrees, and 2 had doctorates. Seventeen were Portuguese, and six were Brazilian. Eighteen were nulliparous, 3 had one child, and 2 had two children. Seventeen had never attended childbirth preparation programs, while 6 had prior experience. As shown in Table 2, four group interviews (GI) were conducted: one with 3 participants, one with 5, and two with 6. Three individual interviews (II) were also conducted. Group interviews averaged 67 min, while individual interviews averaged 11 min.

**Table 2:** Characteristics of participants

Interview	Duration (min)	Partipants (*n*)	Mean age, years (range)	Academic qualifications	Nationality	Gestational age, weeks (range)	Parity (*n*)	Previous childbirth preparation
GI1	60	3 (P1-P3)	37.0 (35–40)	Master’s degree (3)	Portuguese (3)	26–33	0 (2) 2(1)	Yes (2) No (1)
GI2	59	6 (P4-P9)	32.8 (29–36)	High school (1) Graduated (4) Master’s degree (1)	Portuguese (4) Brazilian (2)	28–32	0 (5) 1 (1)	Yes (2) No (4)
GI3	51	5 (P10-P14)	33.6 (28–41)	High school (2) Graduated (2) Master’s degree (1)	Portuguese (3) Brazilian (2)	30–32	0 (5)	No (5)
GI4	98	6 (P15-P20)	31.5 (27–40)	High school (1) Graduated (2) Master’s degree (3)	Portuguese (4) Brazilian (2)	27–33	0 (3) 1 (2) 2 (1)	Yes (2) No (4)
II1	11	1 (P21)	32	Doctorate	Portuguese	30	0	No
II2	12	1 (P22)	40	Doctorate	Portuguese	28	0	No
II3	10	1 (P23)	39	Graduated	Portuguese	30	0	No

### Main themes

**Fig. 1:**
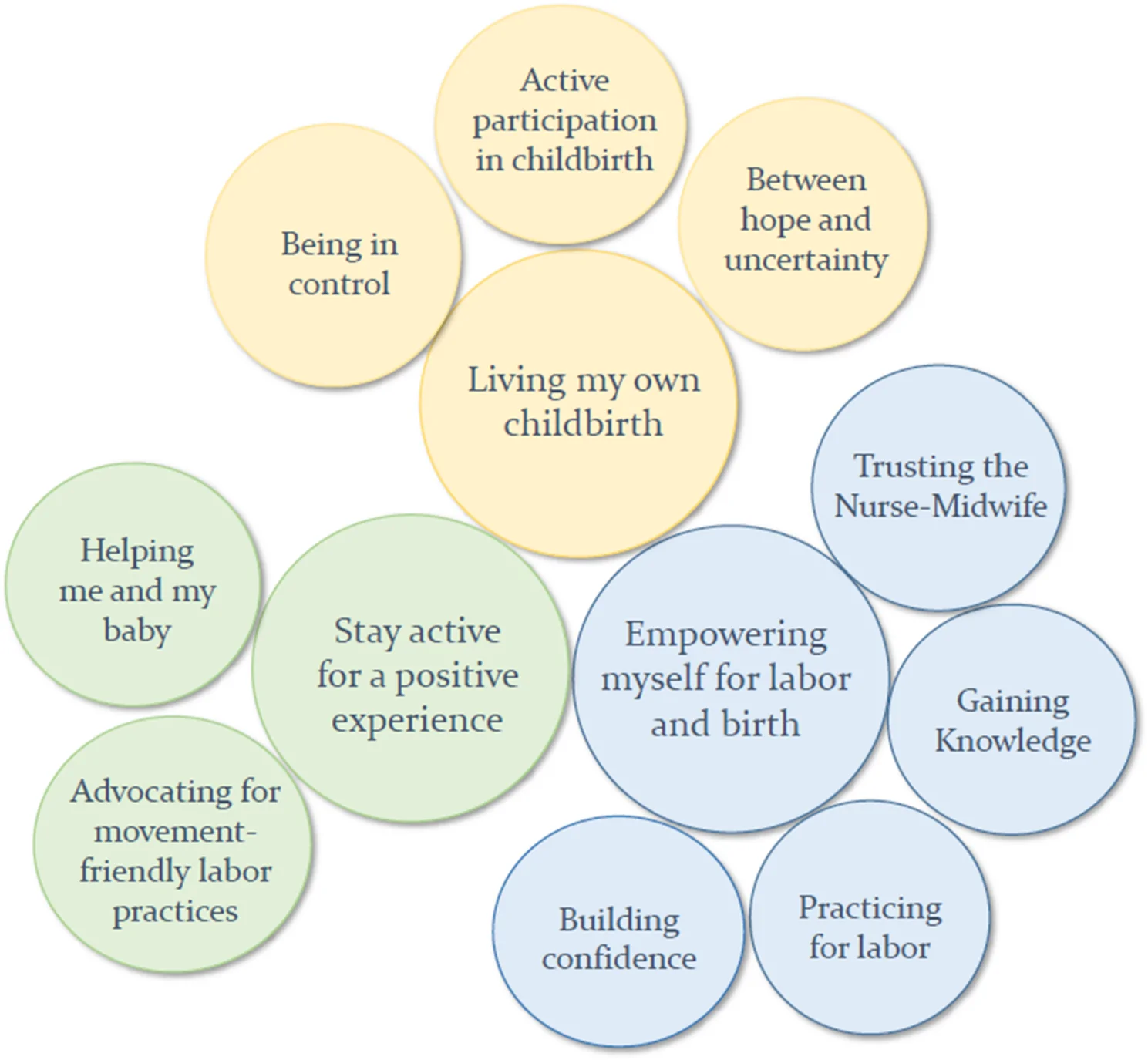
Schematic representation of the themes and sub-themes

The participants’ discourse revealed three main themes: (1) Living my own childbirth; (2) Stay active for a positive experience; and (3) Empowering myself for labor and birth. Figure 1 provides an overview of the main themes and their relationships, offering a visual framework for the presentation of the results.

#### Living my own childbirth

This theme captures how women described their desire to experience childbirth as a personal, meaningful, and self-determined process, shaped by their expectations, values, and previous experiences. Participants articulated how they envisioned their labor and birth experiences. The sub-themes identified were: Being in control; Active participation in childbirth; and Between hope and uncertainty when anticipating childbirth.

##### Being in control

Women described a clear preference for vaginal childbirth, frequently referring to it as “natural,” “normal,” or “vaginal,” and associating this preference with a sense of bodily integrity and an easier recovery.

*“I want a normal birth because*,* from what I’ve seen and heard from others*,* the recovery is easier.” (P17)*

Participants also emphasized the importance of allowing the body to follow its own course during labor, which included respecting bodily signals and delaying the use of epidural analgesia when possible.

*“Ideally*,* it’s a minimally instrumentalized birth*,* meaning we respect all the signals of our body and wait for all the reactions (…) is about taking my time (…) it’s the oxytocin working naturally” (P1)*

*“Maybe avoid or delay the use of the epidural as much as possible and try to listen to the body and see which path it guides us to.” (P13)*

To maintain a sense of control, participants acknowledged their vulnerability during labor and stressed the importance of a supportive environment, with empathetic professionals who could foster a calm and reassuring atmosphere.

*“The environment is something I think about a lot… I would like it to be calm*,* as peaceful as possible*,* with only the necessary people present.” (P1)*

At the same time, women described an ambivalence between wanting to remain in control and recognizing moments in which they might need guidance and support from healthcare professionals.

*“I hope the team on the day is kind*,* welcoming*,* and calm*,* that they inspire confidence*,* and that I feel I can trust them. If I find myself in a situation where I say*,* ‘I’m too tired to decide*,* I don’t care*,* I can’t do anything more. (laughs) I need to feel that I don’t have to be the one in control…’” (P7)*

Another participant suggested that staying at home for as long as possible could be a strategy to retain a sense of control, perceiving the home environment as calmer and less pressurized than the hospital setting.

*“It’s more about being here and feeling calm (…) for me*,* it’s more about feeling calm and not being under the pressure of healthcare professionals.” (P6)*

Some women described ambivalence regarding the use of epidural analgesia, expressing a desire to rely on non-pharmacological strategies and movement, while also feeling pressured by the unpredictability of access to epidural analgesia and the fear of losing control over the timing of the decision.

*“Using the birth ball for certain movements*,* using the shower if needed*,* being allowed to walk*,* but who can guarantee me*,* at that moment*,* how long I can hold out? If I need an epidural*,* the anesthetist might be available*,* but they could also be leaving soon*,* and it’s a case of ‘get it now or you won’t get it at all.” (P2)*

Concerns about episiotomy were also described, particularly by women with previous birth experiences. Engaging in physical activity or pelvic physiotherapy during pregnancy was perceived as a way of preparing the body and regaining a sense of control.

*“I also want to see if I can avoid being cut again. (…) I’ve been doing a lot of exercise*,* going to Pilates classes and such*,* to see if it helps when the time comes*,* at least with the cutting part. But we never know*,* right?” (P19)*

*“I’ve just started doing pelvic physiotherapy*,* and I didn’t realize my scar still hurts… So*,* I’m already trying to prepare my body to avoid being cut again.” (P16)*

##### Active participation in childbirth

Active participation in childbirth was closely linked to women’s autonomy in decision-making and their confidence in managing early labor, often at home.

*“I’d like to stay at home until I’m about 8 centimeters dilated (laughs)*,* then head to the hospital*,* get there*,* and have the baby.” (P6)*

At the same time, women emphasized the importance of flexibility and safety, recognizing that medical interventions, including cesarean birth, might become necessary to protect maternal or fetal wellbeing.

*“If any problem comes up and I suddenly need to have a cesarean*,* that’s totally fine*,* no drama.” (P22)*

Women with previous childbirth experiences reflected on past births, identifying aspects they wished to repeat or change. Being able to actively participate in the birth process was consistently described as a positive experience they hoped to replicate.

*“I hope it will be the same this time*,* with mobility. I only got the epidural when I was in the labor unit*,* and then everything happened very quickly. I felt the contractions*,* knew when they were coming. My husband was by my side*,* and I said to him*,* ‘A contraction is coming*,* right?’ and he said*,* ‘Yes.’ So*,* I felt everything*,* just not the pain as intensely.” (P16)*

Conversely, experiences characterized by limited bodily involvement and reduced decision-making were described as negative and motivated a desire for a different experience in the current pregnancy.

*“I had an epidural and was completely numb from the waist down (…) It’s hard to put into words. I saw him being born*,* but I didn’t feel like I was part of the process. (…) I was there*,* my body was there*,* but I didn’t participate in the birth.” (P4)*

One participant also expressed a desire to choose her birthing position and avoid interventions experienced previously, such as fundal pressure and episiotomy.

*“I’m having my second child*,* and I hope this birth will be a bit different (…) I also want to choose a different birthing position because the last one was uncomfortable*,* and pushing was harder. One of the maneuvers the doctor did was to push on my belly*,* and that was very difficult for me. I couldn’t push*,* I couldn’t breathe*,* and I kept saying*,* ‘Let go of me because I can’t do it like this…’” (P16)*

##### Between hope and uncertainty when anticipating childbirth

Women’s expectations of childbirth ranged from hopeful and positive anticipation to ambivalence and deliberate emotional distancing. Some women described consciously cultivating positive thoughts and visualizing a positive birth experience.

*“I’ve been focusing on positive thoughts*,* mentally preparing myself*,* and visualizing having a wonderful birth…” (P6)*

Others acknowledged childbirth as a challenging and unpredictable experience, expressing hope that it would nonetheless be manageable.

*“I think it will be a mix of pain and unpleasant things (…) I think*,* or I hope*,* that some things will make up for the others. I have the idea that it won’t be an easy process…” (P23)*

For some women, avoiding thinking about childbirth altogether emerged as a coping strategy, described as a way of protecting themselves from unmet expectations.

*“I haven’t consciously thought about it yet*,* to avoid creating expectations…” (P1)*

Across accounts, childbirth was described as a deeply personal and family-centered experience that should be supported by empathetic healthcare professionals. Supportive relationships, partner presence, and immediate skin-to-skin contact were described as central to a positive birth experience.

*“In my first childbirth*,* I was really happy; it was perfect. The nurse-midwives were so delicate (…) I had the most wonderful experience in the world. Right after the birth*,* my son was placed skin-to-skin with me. (…) I didn’t feel any pain because I was so amazed. It was all incredible. The second was really bad; it was an elective cesarean and it was really sad (…) I think the cesarean had a big impact on me*,* the milk coming in*,* I think now*,* looking back*,* it was all stress from being sad*,* from not being well and also the issue of the father not being able to be there.” (P3)*

#### Stay active for a positive experience

This theme reflects how women understood mobility and upright positions during labor as central to a more positive childbirth experience, closely linked to comfort, bodily awareness, and trust in physiological processes. Participants also recognized that, despite this understanding, support for mobility and upright positions was perceived as inconsistent within hospital settings. The sub-themes identified were: Helping me and my baby and Advocating for movement-friendly labor practices.

##### Helping me and my baby: “movement so the baby can follow the right path to be born”

Women described varying levels of knowledge about mobility and upright positions during labor. While many were aware of potential benefits and frequently associated them with the use of a birthing ball, others, particularly nulliparous women, acknowledged limited or absent knowledge on this topic.

*“I confess my complete ignorance on the subject.” (P22)*

Participants described mobility during labor as a way to enhance comfort, by enabling them to adopt positions that alleviated pain, and as a strategy for distraction, self-regulation, relaxation, and supporting labor progress.

*“I find it very reassuring (…) I think it adds a sense of naturalness and*,* almost*,* not quite a home-like environment*,* but it does free us up a bit knowing we can be in almost any position as long as it’s comfortable for us.” (P7)*

*“I think the freedom of movement*,* perhaps… this is a layperson speaking*,* speeds up labor. Maybe it helps minimize the pain and allows for a quicker labor and birth compared to just lying down and waiting… I think it also distracts us from the pain*,* the fact that we are free to move*,* and not just lying there*,* enduring it.” (P10)*

Maintaining freedom to choose positions was described as essential for preserving both comfort and a sense of control.

*“I see childbirth as an experience of freedom*,* allowing me to do whatever makes me feel most comfortable in the moment… Initially*,* being upright gives me a sense of comfort. But if I get tired and exhausted to the point where I want to lie down*,* I want the freedom to do that too.” (P12)*

*“I was always walking back and forth; it was a way to pass the time… I can’t imagine being stuck in a bed*,* unable to do anything during dilation… I’d go crazy!” (P3)*

Mobility was also understood as a way to facilitate optimal fetal positioning within the maternal pelvis, by allowing pelvic movement and the effects of gravity to support labor progression.

*“It allows the baby to settle into the most suitable position*,* right? Movement helps the bone structure to widen*,* so the baby can follow the right path to be born.” (P13)*

In addition, mobility was occasionally associated with the prevention of perineal trauma.

*“What I know is that it can prevent and avoid some severe perineal tears.” (P2)*

Participants described several practical ways of remaining mobile during labor, including using a birthing ball, walking, and taking showers, which were also perceived as effective non-pharmacological strategies for pain relief.

*“I’ve heard some mothers talk about doing some preliminary work instead of the traditional lying down and enduring contractions. They mentioned having some mobility*,* specifically doing exercises*,* working with the birth ball*,* and regularly taking showers… There are alternatives to just lying down and waiting for things to happen*,* and it seems to help.” (P23)*

Women’s appreciation of mobility and upright positions often stemmed from previous positive birth experiences or stories shared by friends, reinforcing their perceived value.

*“The second time was totally different. I stayed home*,* went about my life*,* rested a bit*,* and even walked while feeling contractions. If we keep ourselves distracted*,* we can manage*,* if we keep ourselves occupied*,* time passes*,* and we’re not focused on the pain.” (P15)*

*“I used the ball*,* walked in the corridors*,* and took showers. (…) it helped me a lot.” (P16)*

Despite recognizing these benefits, some women expressed uncertainty about their ability to remain active during labor, particularly due to concerns about coping with pain.

*“I think what we’ve talked about is very important (…) But honestly*,* I don’t know… putting it into practice*,* I think it would really require a lot of mental preparation*,* and I’m not sure if I wouldn’t change my mind once I got there. I think I might not be able to do it.” (P19)*

##### Advocating for movement-friendly labor practices

Although women valued mobility and upright positions, they expressed concerns about whether these practices would be supported in hospital settings. Participants perceived that the feasibility of remaining active during labor depended largely on the attitudes and openness of healthcare professionals. Women described feeling vulnerable during labor and feared that, in this context, they might comply with restrictive practices if professionals did not actively encourage mobility.

*“For me*,* this is extremely important. But how much this will be respected… I don’t know…” (P1)*

*“For me*,* this is extremely important. But how much this will be respected or feasible… I don’t know*,* which is why I try not to have too many expectations. (…) It’s important for us to empower ourselves*,* but it’s also crucial for healthcare professionals to start being open to this.” (P1)*

Participants highlighted a tension between the need to empower themselves and the responsibility of healthcare systems and professionals to prioritize women-centered, evidence-based care.

*“I think it depends on whether you’re lucky enough to get a professional who allows it because sometimes you’re so vulnerable that you’ll accept whatever the professional says.” (P9)*

#### Empowering myself for labor and birth

This theme reflects how women described empowerment as a process developed during pregnancy, particularly through participation in childbirth preparation programs, combining trust in professionals, access to knowledge, practical preparation, and growing self-confidence. The sub-themes identified were: Trusting the nurse-midwife; Gaining knowledge; Practicing for labor; and Building confidence.

##### Trusting the nurse-midwife

Women described feeling more secure when childbirth preparation was facilitated by a nurse-midwife, whom they perceived as a reliable source of evidence-based information. This trust was often contrasted with uncertainty about information obtained from social media or informal sources.

*“I try to avoid searching on social media as much as possible because not everything we see and are advised on is accurate. In this sense*,* I feel much more secure with the nurse-midwife who is there with us.” (P10)*

Participants also valued the nurse-midwife’s familiarity with local hospital practices, care philosophy, and available resources. This contextual knowledge was seen as helping women adjust expectations and prepare more realistically for childbirth.

*“I think it is very beneficial for us to take this course with someone from the area*,* from the hospital*,* or at least someone who has direct contact with the hospital and knows how it works. This helps us adjust our expectations.” (P7)*

##### Gaining knowledge

Women consistently expressed the need to gain knowledge in order to feel more prepared and capable of participating in decision-making during labor. They highlighted interest in learning about mobility and upright positions, their potential benefits, and the scientific evidence supporting different options.

*“First*,* I would like to know what the other alternatives are*,* and then to understand the advantages and disadvantages. I don’t know if there are studies on this or not; I don’t know if it’s something new. If a woman has more issues in a certain position*,* if she tears more*,* if she has to be cut*,* if there’s any relationship between the position and these outcomes.” (P21)*

Participants emphasized that knowledge provided practical tools for managing labor, helping them remain focused and actively engaged.

*“I want to feel that I have the tools to manage my labor. I may need support and not do it all alone*,* but I want to feel that I am actively contributing.” (P4)*

*“When we’re in pain*,* whether it’s day or night and regardless of other conditions*,* knowing what to do is very helpful. Being able to focus on something makes a big difference and helps a lot.” (P3)*

Understanding hospital practices and available resources was also described as important for planning and feeling prepared.

*“Being aware of what might be available at the place where we will give birth (…) knowing what resources will be at our disposal and being informed about different possibilities*,* so we can choose what makes us feel most comfortable and what makes the most sense at the moment.” (P13)*

Some women highlighted the importance of sharing this knowledge with their partners, enabling them to offer more effective support during labor.

*“I think it’s important for couples to do this together. It’s crucial for them to know what we’re working on and how they can help*,* and the role they can play.” (P18)*

Knowledge about women’s rights during labor was also described as central to feeling confident in refusing unwanted interventions.

*“Knowing what we can allow and not allow*,* knowing what our rights are*,* having that knowledge*,* from the exams to*,* the breaking of membranes*,* because if they are going to intervene*,* we need to be informed.” (P2)*

*“There’s the famous drip. Am I obliged to accept it*,* the oxytocin*,* or can I refuse it? Those things… having a bit more knowledge so that we can make informed decisions.” (P9)*

At the same time, women expressed ambivalence regarding the amount of information received. While many valued detailed knowledge, others acknowledged that excessive information could increase anxiety and pressure when making decisions.

*“The more detailed the information*,* the better. In my case*,* as a first-time mother who hasn’t experienced anything like this yet*,* I’m completely blank*,* and I think I’ll absorb everything I hear.” (P22)*

*“It’s important to know some key points about labor and what’s going to happen. But I also think it’s important not to have too much information because sometimes having too much knowledge creates a lot of anxiety and the feeling that we have to decide everything.” (P7)*

Despite this ambivalence, women recognized that being informed enhanced their ability to participate meaningfully in decision-making, even when it generated some anxiety.

*“The more informed we are*,* yes*,* it definitely creates more anxiety. If we don’t know anything*,* we just go*,* and whatever happens*,* happens. But as we get more information*,* even though it may cause more anxiety*,* I also feel we can have a say. We go in with some backup*,* some prior information that allows us to have an opinion. Because the truth is*,* if we have no information*,* no matter how much the team includes us and helps us participate*,* we won’t be able to make any informed decisions.” (P18)*

##### Practicing for labor

Women described practical, experiential learning as essential for translating knowledge into action during labor. Hands-on training during group childbirth preparation sessions was particularly valued, as it allowed women to explore movements, positions, and bodily responses in advance.

*“In that moment*,* with anxiety and pain*,* if it’s just theoretical knowledge*,* it isn’t as automatic. When we think about anxiety in different contexts*,* we do what we’ve practiced*,* not just what we know in theory. At least we start to find the movements and positions we’re most comfortable with*,* so when the time comes*,* we have an almost automatic reflex to find them*,* rather than trying for the first time to find a position we’ve never worked on.” (P18)*

Participants also emphasized the importance of involving partners in practice, so they could provide effective support during labor.

*“We should practice at home*,* gaining that experience*,* and our partner should also know what helps us the most and what relieves our pain. Having someone to rely on who already knows what is most comfortable and what helps the most is crucial*,* especially if we can’t think quickly at the moment.” (P20)*

Women believed that having practiced strategies in advance would help them remain active and engaged during labor, even in longer or more demanding situations.

*“If we don’t have these tools*,* and it’s a longer labor*,* at some point*,* from an anxiety and stress perspective*,* we’ll just be watching the clock*,* thinking*,* ‘Why is this taking so long?’ So*,* I think it’s very important to keep ourselves distracted. Even if it doesn’t make a difference in the labor itself*,* it makes a huge difference for us and how we perceive that moment if we can stay engaged with some options.” (P7)*

*“Having our techniques to try to enter our own world*,* that may or may not be used on the day*,* but just knowing about them is great. It gives me peace of mind to know that I have techniques I can rely on during labor to calm me down.” (P1)*

##### Building confidence

Women described confidence as emerging from the combination of knowledge, practice, and supportive relationships. Feeling informed enabled them to communicate more effectively with healthcare professionals and to participate actively in decisions during labor.

*“Feeling more comfortable and having more knowledge so that when the time comes*,* I can confidently say ‘no’ or ‘yes’ when things happen. I want to feel prepared to say*,* ‘Can we do this another way? I understand it’s usually done this way*,* but can we try something different?‘” (P4)*

Confidence was also associated with understanding different labor options and becoming familiar with the birth environment.

*“I believe a well-informed woman will be much more confident.” (P21)*

*“If we don’t have information*,* we won’t be able to make informed decisions. It’s almost like playing a guessing game*,* which is not what we want (…) and what best suits us*,* because sometimes there is no clear right or wrong.” (P18)*

Effective communication with the healthcare team, supported by professional competence and empathy, was described as crucial for maintaining confidence.

*“Not being afraid to ask questions (…) understanding what is being done and why it has to be done that way.” (P4)*

At the same time, women emphasized that empowerment depended not only on their own preparation but also on a clinical environment that respected their preferences and invited their participation.

*“As women*,* we need to empower ourselves and recognize that the birth experience is ours. We should have all the necessary information*,* but it’s also crucial that healthcare professionals give us the opportunity to be involved.” (P1)*

Some women described self-confidence as an internal resource they were actively developing, while others already perceived themselves as confident in managing labor.

*“I feel quite confident. I’m naturally calm*,* and I think I know how to handle pressure and chaos a bit*,* and I have a high pain tolerance. So*,* I think when the time comes*,* I’ll be ready to face whatever happens (laughs). Firstly*,* it’s my moment*,* ours. I try to distance myself as much as possible from my surroundings and focus on what’s most important*,* and I think that will help me.” (P10)*

*“It’s about preparing and mentally conditioning myself (…) the more prepared and aware I am*,* and the better I can self-regulate*,* the more easily I’ll handle and distance myself from any difficulties I might encounter. Of course*,* it also requires my own effort.” (P13)*

Finally, sharing experiences with other women during group preparation sessions was described as enhancing confidence and providing emotional reassurance.

*“Various women*,* each with their doubts*,* I think it works almost like a support group. When so many doubts overwhelm us and we start researching*,* which can make us anxious*,* it feels almost liberating to be in a sharing environment. I think that’s important*,* and I believe it’s therapeutic in itself.” (P7)*

## Discussion

The analysis highlighted a strong desire among women to experience labor and birth as deeply personal and autonomous processes, with a sense of control and active participation. Many women turn to childbirth preparation programs led by midwives as a means of empowerment, seeking to enhance their knowledge, skills, and confidence. In this context, the freedom to move during labor is seen as a critical strategy. However, while women recognize that empowerment is key to a positive birth experience, they also understand that it is not sufficient if clinical environments fail to meet their expectations, which can lead to dissatisfaction and frustration.

### My birth is mine

Most participants anticipated a vaginal birth, considering it a physiological option aligned with their understanding of childbirth processes. They strongly desired active involvement in childbirth decisions, respecting bodily signals, taking their time, and allowing oxytocin to work in a calm environment. Staying home during early labor was seen as a way to maintain control and avoid pressures, such as premature use of epidural analgesia. These findings align with research [28], which highlighted women’s preference for physiological childbirth. Despite childbirth’s unpredictability, women sought a sense of achievement and control, even when medical interventions became necessary. Other studies confirm that most women in high-income countries prefer vaginal birth, despite high levels of medicalization [29, 30].

While women desired control over their childbirth experience, they also felt the need to trust healthcare professionals who understood and supported this ambivalence. This duality was evident in women’s perceptions of childbirth [31], where they wanted to be active decision-makers while also relying on their partner and nurse-midwife for support when feeling vulnerable. Acknowledging this ambiguity can enhance the childbirth experience by validating women’s expectations and valuing their voices.

Women with previous childbirth experiences valued being active participants and holding their baby immediately after birth, while interventions such as fundal pressure (Kristeller manoeuvre) were recalled as distressing when women felt uninvolved and powerless. It is important to note that the Kristeller manoeuvre is not supported by current evidence and is considered an inappropriate intervention in many international guidelines [1]. Although not recommended, its mention in women’s accounts suggests that this practice may still occur in some clinical contexts, reflecting variability between evidence-based recommendations and actual intrapartum care, rather than endorsed practice. Labor was generally anticipated as a positive experience, especially by nulliparous women, even when perceived as challenging. This reflects a sense of coherence, where labor is perceived as manageable through available resources and flexible decision-making, enhancing resilience and positivity.

A strong sense of coherence leads to a positive, relaxed focus on the baby, while a weaker sense may result in anxiety focused on the woman’s effort during labor. This coherence can be shaped by childbirth preparation [32]. However, previous negative experiences often led to frustration when medical systems did not support women’s autonomy, contributing to disillusionment. This may explain why nulliparous women expressed greater trust in childbirth, as they had not yet encountered such dissonance. To foster trust and self-confidence, hospital environments must implement structural changes that support physiological processes and respect women’s autonomy [1].

All participants with previous childbirth experiences described aspects they wished to replicate or change in future labors. These memories, whether positive or negative, significantly influenced self-confidence and self-esteem as women and mothers. Satisfactory experiences were recalled as meaningful and affirming [33], while negative ones could become a source of distress [34].

Midwives play a crucial role in shaping these impactful memories during pregnancy and labor. Participants valued active involvement in vaginal birth, empathetic care, and immediate skin-to-skin contact, which symbolized the reward of their efforts. These findings align with WHO recommendations for a positive childbirth experience [1], defined as one that meets or exceeds women’s expectations through respectful, continuous, and competent support. Negative experiences to be avoided included lack of participation, excessive interventions, and a sense of lost control. A systematic review reported negative childbirth experiences ranging from 6.8% to 44%, often associated with unmet expectations, insufficient support, and interventions such as instrumental or cesarean births [35].

In this study, most participants understood and valued mobility and upright positions during labor, recognizing them as essential for supporting labor progression and pain management. Freedom to move was perceived as a fundamental right, central to comfort and control, with the ability to choose positions linked to more positive experiences [28]. A meta-synthesis showed that preferences for birthing positions are shaped by personal and cultural beliefs. In Europe, women often adopt horizontal positions due to perceived risks or healthcare professionals’ recommendations [2]. A Danish study found that over 80% of women did not achieve their preferred birthing position, with the culture of the birth setting and professionals’ attitudes playing a significant role [16]. Participants’ understanding of mobility as a coping strategy should be encouraged rather than constrained. Despite valuing mobility, women described challenges related to pain management, sometimes leading to frustration. In highly medicalized environments, routine immobilization, limited support, and stressful conditions may interfere with oxytocin release, negatively affecting pain management and maternal physiology [36]. Unsurprisingly, some multiparous women in this study expressed doubts about their ability to cope with labor. Pain management strategies learned during childbirth preparation need to be supported intrapartum, within environments that respect childbirth physiology and provide continuous midwife support. Past experiences shape a woman’s self-efficacy for childbirth, with positive experiences reinforcing perceived capability and negative ones undermining it [37]. Promoting self-efficacy involves pain management training and positive reinforcement within supportive environments, helping women remain focused between contractions [38]. Participants emphasized the need for skill development through body awareness and practical strategies, ideally involving partners. This empowerment enhanced confidence and supported decision-making during labor. An Australian study similarly found that experiential learning fostered a sense of control and capability [39]. Participants also valued social connections with other women as a source of reassurance. A study in England reported that meeting others in similar situations was a key motivation for attending childbirth preparation programs, helping normalize concerns and foster supportive relationships [19]. For midwives, facilitating these social interactions supports the development of self-efficacy by creating safe spaces for shared learning [21].

### Promoting active and supportive labor environments

Beyond wanting to remain active during childbirth, many participants perceived that freedom of movement was still uncommon in hospital settings. They anticipated having to comply with healthcare professionals’ requests due to the vulnerability associated with labor, often resulting in frustration. A study in Ireland reported similar concerns, noting that while women trusted professional expertise, they also sought experiential knowledge from other women. However, they often lacked confidence to challenge biomedical routines and therefore complied with them [40]. These findings underscore the importance of recognizing and valuing women’s expectations during childbirth preparation and implementing interventions that support their agency [3].

Participants acknowledged that empowering women during pregnancy is essential, but its impact remains limited if hospital settings, particularly public ones, do not support women’s expectations. This uncertainty undermines adequate preparation for childbirth and should be addressed antenatally. Women expressed a desire for healthcare professionals to engage with their birth plans. Rather than attributing support or resistance to specific professional groups, these findings point to the influence of organizational cultures, institutional routines, and models of care. Hospital environments strongly shape women’s ability to make decisions, which improves when they feel respected and when their birth plans are acknowledged. These findings align with a Spanish study [20], which showed that an intervention promoting shared decision-making did not increase birth plan submission but reduced epidural use and encouraged alternative pain relief methods. Barriers included power imbalances, negative attitudes, and rigid institutional policies. To support women’s active participation during labor, childbirth preparation should strengthen knowledge and self-efficacy while being matched by organizational change. One example is the *Labour Hopscotch Framework*, developed within a midwifery-led model of care in Ireland, which was associated with a substantial reduction in epidural use [41]. Such approaches highlight the importance of aligning antenatal education with supportive intrapartum environments.

It is essential to support women in making autonomous and satisfactory decisions during childbirth. Antenatal education and hospital practices should prioritize recognizing and valuing women’s expectations. In this study’s context, midwife-led intrapartum care was perceived as crucial for respecting women’s choices. Hospitals should adopt person-centered approaches that promote privacy, calm, and flexibility in birthing rooms, enabling women to remain active and adapt to their preferences. This supports the personal, intimate, and family-centered childbirth experience that women described as meaningful.

### Strengths and limitations

This qualitative study offers an in-depth exploration of pregnant women’s knowledge, expectations, and needs regarding mobility and upright positions during labor, with a focus on decision-making as a contextual and relational process. While prior research has largely examined preferences or beliefs using survey or intervention designs, this study adds qualitative insight into how women make sense of available options and anticipate their participation in decisions within real care contexts. The combination of group and individual interviews strengthened the study by allowing both shared reflection and more private individual accounts, thereby enhancing the depth and breadth of the data. The inclusion of nulliparous and multiparous women from diverse nationalities, educational backgrounds, regions, and primary care units enriched the findings and supported transferability. Despite the online format, group interviews were characterized by active engagement and rich discussion. To enhance credibility and dependability, data analysis involved collaborative interpretation and reflexive discussion among the researchers.

However, some limitations should be acknowledged. The use of both group and individual interviews may have influenced how participants expressed their views, as each format creates different conditions for sharing experiences, expectations, and uncertainties. In addition, the findings should be interpreted within the specific context in which the study was conducted.

## Conclusion

This study shows that pregnant women value mobility and upright positions as part of an active and participatory birth experience, but they need evidence-based information, practical preparation, and supportive professional care to feel confident in using these options during labor.

Childbirth preparation programs should therefore include strategies that strengthen women’s self-efficacy and decision-making capacity, rather than focusing only on information provision. The findings also point to the need for greater alignment between antenatal preparation and hospital-based labor and birth care, so that women’s preferences for mobility and active participation can be supported in practice. Future research should develop and evaluate interventions that promote informed and supported decision-making about mobility during labor.

## Data Availability

The datasets generated and analyzed during the current study are not publicly available due to the qualitative nature of the data and the risk of compromising participant confidentiality but are available from the corresponding author on reasonable request.
